# Phase Behavior and Structure of Poloxamer Block Copolymers in Protic and Aprotic Ionic Liquids

**DOI:** 10.3390/molecules28217434

**Published:** 2023-11-05

**Authors:** Aikaterini Tsoutsoura, Zhiqi He, Paschalis Alexandridis

**Affiliations:** Department of Chemical and Biological Engineering, University at Buffalo, The State University of New York (SUNY), Buffalo, NY 14260-4200, USAzhiqihe@buffalo.edu (Z.H.)

**Keywords:** poly(ethylene oxide), poloxamer, ionic liquid, lyotropic liquid crystal, phase behavior, scattering

## Abstract

Ionic liquids are promising media for self-assembling block copolymers in applications such as energy storage. A robust design of block copolymer formulations in ionic liquids requires fundamental knowledge of their self-organization at the nanoscale. To this end, here, we focus on modeling two-component systems comprising a Poly(ethylene oxide)-poly (propylene oxide)-Poly(ethylene oxide) (PEO-PPO-PEO) block copolymer (Pluronic P105: EO_37_PO_58_EO_37_) and room temperature ionic liquids (RTILs): protic ethylammonium nitrate (EAN), aprotic ionic liquids (1-butyl-3-methylimidazolium hexafluorophosphate (BMIMPF_6_), or 1-butyl-3-methylimidazolium tetrafluoroborate (BMIMBF_4_). Rich structural polymorphism was exhibited, including phases of micellar (sphere) cubic, hexagonal (cylinder), bicontinuous cubic, and lamellar (bilayer) lyotropic liquid crystalline (LLC) ordered structures in addition to solution regions. The characteristic scales of the structural lengths were obtained using small-angle X-ray scattering (SAXS) data analysis. On the basis of phase behavior and structure, the effects of the ionic liquid solvent on block copolymer organization were assessed and contrasted to those of molecular solvents, such as water and formamide.

## 1. Introduction

The self-assembly of amphiphiles, such as surfactants, lipids, and block copolymers, constitutes an area of cornerstone molecular engineering research and offers an effective and efficient strategy for manufacturing complex nanostructures and novel materials [1]. In the case of block copolymers, microphase separation in bulk polymers [2] or solutions [3,4] is driven by the incompatibility of the different blocks that are restricted spatially by their chemical connection. The type of structure (spheres, cylinders, and lamellae with characteristic scale lengths in the order of 10–100 nanometers) obtained by self-assembly depends on the volume fraction of one type of block compared to the other [2,5]. Solvents that are selective for one block can amplify the self-assembly of block copolymers and modulate the type of structure formed [6,7,8,9]. Self-assembled block copolymers are of significant importance in various formulations [10], the nanoscale patterning of surfaces [11], nanomaterial synthesis [12], and drug delivery carriers [13,14].

Ionic liquids (ILs) are salts that are liquid at ambient conditions and consist of organic cations and inorganic or organic anions [15,16]. They possess interesting physicochemical properties, including negligible vapor pressure, good thermal stability, high ionic conductivity, broad electrochemical potential windows, good solubility, and high synthetic flexibility [17,18]. Their liquid interionic structure and dynamics are responsible for many of their interesting physicochemical properties [19]. Ionic liquid solvents can facilitate the formation of the self-assembled structures of various amphiphiles and block copolymers [20,21,22,23] and find potential applications in diverse fields such as electrochemistry [24,25,26], separations [27,28], biomass processing [29,30], catalysis [31], drug delivery [32], and space technology [33].

Poly(ethylene oxide) (PEO)-based block copolymers comprising blocks of different polarity to PEO, such as poly (propylene oxide) (PPO), are of great interest due to the aqueous solubility and biocompatibility of the PEO moiety and their thermoreversible self-assembly [4]. In recent years, solid PEO polymers have been widely studied for lithium battery applications due to their alkali ion conductive properties [34,35]. The self-assembly of PEO-based amphiphilic block copolymers, in particular, PEO-PPO block copolymers that are commercially available as Poloxamers and Pluronics, has been reported, and their phase behavior and nanostructure in dilute and concentrated aqueous solutions have been extensively characterized [36,37]. The micellization of PEO-PPO block copolymers [36] in dilute aqueous solutions and the formation of lyotropic liquid crystalline (LLC) structures at concentrated solutions are driven by the hydrophobic (or relatively less hydrophilic) PPO blocks [4,37]. In addition to water, which is the prime solvent for PEO-containing amphiphiles, solvents, such as glycols, hydrazine, formamide, and, more recently, ionic liquids, support amphiphile self-assembly [38,39,40].

The solvation power of ionic liquids is related to their chemical structure [41]. The intrinsic structural heterogeneities result in nanosegregation [42]. The low-charge density regions (nonpolar networks composed of alkyl side chains of cations, such as imidazolium and pyrrolidinium and or anions like alkylsulfates and alkylsulfonates) are segregated from the high-charge density nano-domains (polar domains composed of the remainder of anions and cations), giving an amphiphilic character to the ionic liquid [43]. Therefore, ILs have the potential to dissolve polar and apolar solutes in the respective nano-domains [44]. Both protic and aprotic ILs have been shown to promote the self-organization of amphiphilic molecules [45]. Protic ionic liquids (PILs) are formed via the transfer of protons from a Bronsted acid to a Bronsted base, such as a primary amine in a stoichiometric combination [46], and are of significant interest due to their potential use as electrolytes for fuel cells [47]. Ethylammonium nitrate (EAN) is the best-studied protic ionic liquid for self-assembly purposes [48]. Aprotic ionic liquids (AILs) [49] are formed by an organic molecular cation (alkyl pyridinium, dialkylimidazolium, or alkylpyrrolidinium) and anions of oxidic character (PF_6_^−^, BF_4_^−^, CF_3_SO_3_, (CF_3_SO_2_)_2_N, NTF_2_, or (bis(trifluoromethylsulfonyl)imide)) [50]. Of interest here are imidazolium-based AILs, specifically, 1-butyl-3-methyl imidazolium hexafluorophosphate (BMIMPF_6_) and 1-butyl-3-methyl imidazolium tetrafluoroborate (BMIMBF_4_) due to their well-studied structural [51] and physicochemical characteristics [52].

The emerging applications of block copolymers in ionic liquids pertain to lithium batteries and fuel cells [47,53]. Solid-like nanostructured polymer electrolytes can retain the properties of ionic solvents (such as high ionic conductivity) and the mechanical properties of polymers [54]. Other applications are envisioned in the area of biomechanics with electroresponsive gels for biomimetic processes and electromechanical actuators and devices [26,55] that can be designed using ionic liquid polymer mixtures [47]. Further applications of block copolymers and ionic liquids are expected in the area of drug delivery [32,56] and separations [28,57]. Hence, the combination of block copolymers and ionic liquids presents a powerful platform for designing materials of combinatorial diversity [58].

This work addresses the role of ionic liquids as self-assembly media. The interactions of ionic liquid solvents and the amphiphile block copolymer, as reflected in the structural characteristics of self-assembled microstructures, should be fundamentally investigated in order to successfully design functional formulations and composite materials for large-scale applications. To this end, the phase diagrams of the symmetric (50% PEO (solvophilic) and 50% PPO (solvophobic)) PEO-PPO-PEO block copolymer Pluronic P105 (EO_37_PO_58_EO_37_) were mapped out across the whole concentration range using binary mixtures with model protic (EAN) and aprotic (BMIMPF_6_, BMIMBF_4_) ionic liquids. Structural analysis of the different phases formed was undertaken. Our conclusions were drawn pertaining to ionic solvent selectivity and its origins, and comparisons were drawn with molecular solvents. The lyotropic liquid crystalline structure of PEO-PPO-PEO block copolymers in ionic liquid solvents is characterized here for the first time.

## 2. Results and Discussion

### 2.1. Phase Behavior of Binary EO_37_PO_58_EO_37_ + Ionic Liquid Systems

**Figure 1:**
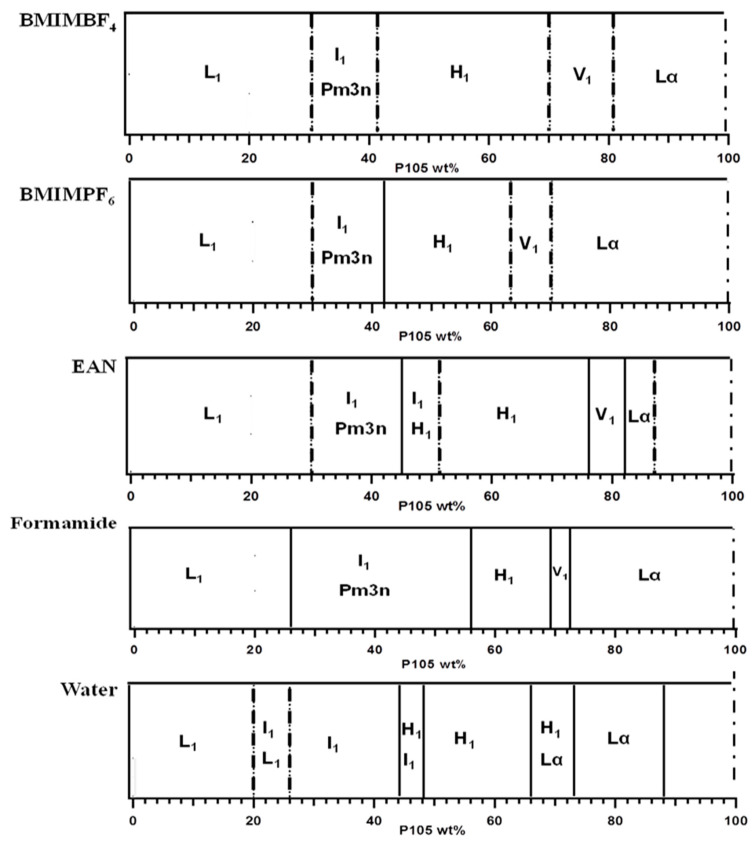
Phase boundaries of the binary systems (EO_37_PO_58_EO_37_) + solvent at T = 25 °C. The vertical lines correspond to phase boundaries. The dotted lines indicate lower accuracy in the determination of the phase boundary. L_1_: micellar phase; I_1_: cubic phase; H_1_: hexagonal phase; V_1_: bicontinuous cubic phase; L_α_: lamellar phase.

The phase diagrams for the binary systems of the Pluronic P105 (EO_37_PO_58_EO_37_) block copolymer and ionic liquid solvents are presented in Figure 1. The phase diagrams are determined based on the sample optical appearance and SAXS diffraction patterns according to the procedure discussed in Materials and Methods. The phase diagrams of Pluronic P105 in water and in formamide are taken from previous works by Alexandridis and co-workers [4,59].

At a low concentration of block copolymer in an ionic liquid, the local interactions determine the formation and structure of the assemblies [22,23]. By increasing the block copolymer concentration, the interaction (repulsion) between the assemblies increases, and ordering takes place [3,4]. The result is rich phase behavior comprised of several one-, two-, or three-dimensional lyotropic liquid crystalline (LLC) structures of different scale lengths. A micellar cubic structure is observed in the 52–67 *v/v*% EAN composition range. At 13–46 *v/v*% EAN, the samples are birefringent (optically anisotropic), indicating lamellar and hexagonal LLCs. Specifically, for the 22–46 *v/v*% EAN concentration range, the hexagonal structure is established based on SAXS, as discussed in the next section. Similarly, SAXS patterns indicate lamellar structures in 13–18 *v/v*% EAN. The same sequence of structures is identified in the binary systems of P105 with the two aprotic ionic liquids considered here. The micellar cubic structures are stable in the range of 53–65 *v/v*% BMIMPF_6_, and the birefringent region is stable within 33–53 *v/v*% BMIMPF_6_. The SAXS analysis confirms the hexagonal region is within the concentration range 33–53 *v/v*% BMIMPF_6_, whereas the lamellar structure is stable below 33 *v/v*% BMIMPF_6_. The micellar cubic region extends up to 66 *v/v*% BMIMBF_4_. Anisotropic structures are identified at 17–56 *v/v*% BMIMBF_4_. Specifically, hexagonal LLC is identified in the range 28–56 *v/v*% BMIMBF_4_, and lamellar LLC below 17 *v/v*% BMIMBF_4_.

The sequence of the LLC phases formed by EO_37_PO_58_EO_37_ is the same for all ionic liquid solvents tested here, and this is consistent with the phase behavior of surfactants and lipids in water [1,60] but with variations to the composition ranges of their stability. For example, the extent of the micellar cubic phase is larger in EAN than in the AILs. In P105 + EAN, we also note some coexistence of micellar cubic with hexagonal microstructures. The hexagonal, bicontinuous cubic, and lamellar LLC phases all start to form at higher pluronic concentrations in EAN compared to the AILs. When comparing BMIMBF_4_ and BMIMPF_6_, the hexagonal phase has a larger range in BMIMBF_4_ than in BMIMPF_6_.

The micellar cubic and hexagonal assemblies formed in the three Pluronic P105 + IL systems have a positive curvature, i.e., the curvature increases away from the polar solvent and bends towards the less polar PPO domains. The bicontinuous cubic and lamellar assemblies have a zero interfacial curvature. No “water-in-oil” structures [61,62] are formed because the ionic liquids selected in this study are not selective for the PPO block.

When comparing the PEO-PPO-PPO block copolymer phase behavior in ILs to that in water or formamide [4,59], the stability region of the L_1_ solution phase in ionic liquid solvents is shifted to higher polymer concentrations compared to that in water or formamide. The increased composition range of stability of the various LLC phases in ionic liquids, as shown in Figure 1, indicates various extents of PEO swelling with the IL solvent. On a molecular scale, the swelling has to do with the preferential location of solvent inside the polymer coils. Later, we will discuss the SAXS results that directly demonstrate ionic liquid solvent selectivity, which allows for an in-depth comparison to water and other organic solvents. In addition, we note that the micellar cubic (Pm3n) phase and the bicontinuous cubic phase are absent in the binary P105 + water system at 25 °C, whereas both form in ionic liquid solvents and in formamide. The concentration range of stability of the micellar cubic phase in ionic liquids is smaller than that in water or formamide.

### 2.2. Structure in the Binary EO_37_PO_58_EO_37_ + Ionic Liquid Systems

**Figure 2:**
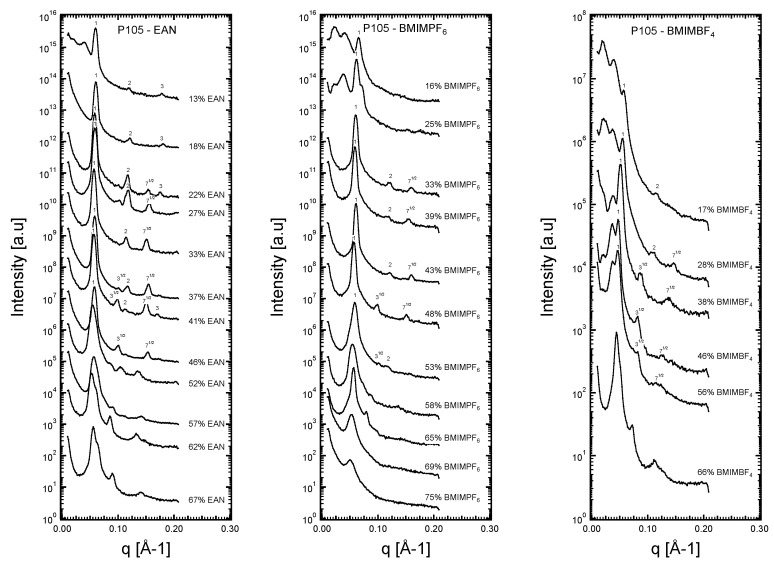
SAXS diffraction patterns obtained from the EO_37_PO_58_EO_37_ + IL single-phase samples across a wide range of concentrations: 13–67 *v/v*% EAN, 16–75 *v/v*% BMIMPF_6_, and (17–66 *v/v*% BMIMBF_4_. The corresponding ratio of each peak to the first and most intense peak is noted (the scattering intensities have been multiplied by an offset factor to facilitate the inspection of the data).

Representative SAXS intensity patterns for each of the Pluronic + IL binary systems are shown in Figure 2. The corresponding ratio of each peak to the first and most intense peak is noted in Figure 2. For the micellar cubic structure, the ratio of the relative position of the Bragg peaks is not displayed in Figure 2, but it will be discussed later.

**Figure 3:**
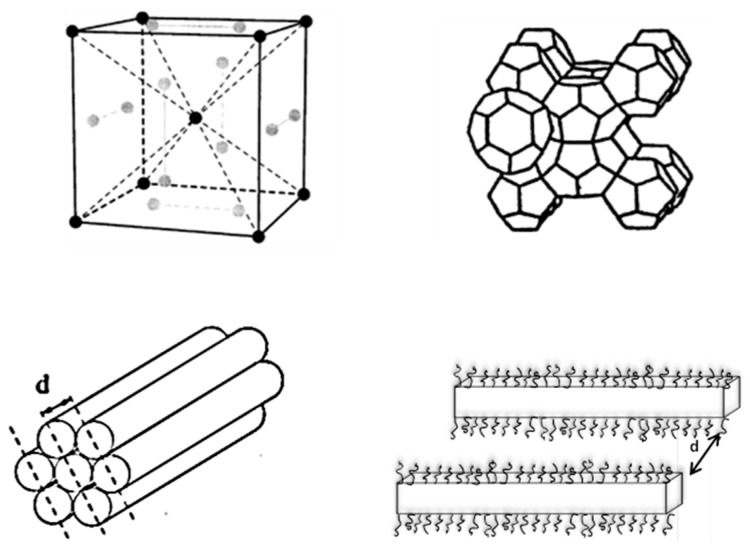
(**Top**) Positions of micelles in the Pm3n unit cell. Polyhedral representation of the structure of the Pm3n cubic phase. Each polyhedron represents a micelle of amphiphile plus its associated solvent. (**Bottom**) Hexagonal-phase cylinders (two-dimensional hexagonal packing of amphiphilic rods) and lamellar phase (one-dimensional stacking of amphiphilic bilayers).

The SAXS intensity patterns show high-order peaks that are particularly intense in the case of EAN. The recorded high-order peaks are weaker and tend to vanish in the case of BMIMPF_6_ and BMIMBF_4_. The samples with peaks that follow the sequence 1:3^1/2^/2/7^1/2^, relative to one of the first and most intense peaks, are assigned the hexagonal LLC structure, in this case, consisting of supramolecular structural elements (i.e., cylindrical block copolymer micelles enclosing the less polar PPO block) that are packed in a hexagonal lattice (Figure 3). SAXS intensities, with peaks obeying the sequence 1:2:3, are assigned to lamellar structures that exhibit one-dimensional periodicity (one-dimensional microstructure consisting of planar assemblies (Figure 3)). As is established later in this section, the micellar cubic structure identified here can be described by two quasi-spherical micelles and six disk-shaped micelles per unit cell, a structure analogous to the packing of polyhedra in cubic clathrate hydrates (Figure 3).

In the Pluronic P105 + ΒΜΙΜΒF_4_ system_,_ shoulders can be present in the SAXS patterns at q-values below the first and most intense peak. We believe the observed spectra irregularities to be the result of the inherent crystallinity of PEO blocks, which is more pronounced as the PEO content increases in the binary mixture. Block copolymers with higher PEO content were studied with the addition of BMIMPF_6_, and the ionic liquid appeared to reduce the crystallinity of the PEO block [63]. However, no significant effect in the crystallinity of the PEO blocks is observed when BMIMBF_4_ is the ionic solvent for the same concentration ranges. This is likely due to favorable hydrogen bond interactions between the imidazolium rings and [BF_4_]^−^ anions over that of PEO. Indeed, after performing SAXS measurements at a temperature higher than the melting point of Pluronic P105, the irregularities in the patterns disappear without changing the specific location of the peaks. That makes us confident about the validity of the structural analysis. In the case of the imidazolium-based ionic liquid with [PF_6_]^−^, these shoulders are not present.

**Figure 4:**
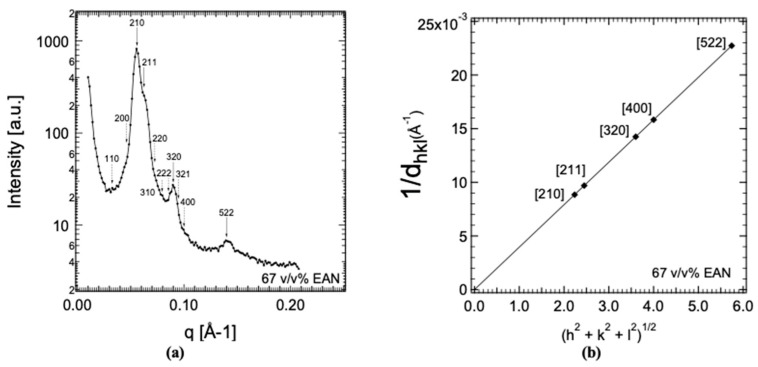
(**a**) SAXS data obtained from the 33:67 vol. % P105 (EO_37_PO_58_EO_37_) + EAN samples. The arrows indicate the indexing of the structure to the Pm3n space group. (**b**) Reciprocal d spacings (1/d_hkl_) of the reflections marked in the SAXS diffraction pattern plotted versus m = (h^2^ + k^2^ + l^2^)^1/2^. The micellar cubic lattice parameter obtained from the slope of the plot is 253 Å.

A representative SAXS diffraction pattern obtained in the LLC phase formed between the solution and the hexagonal LLC phase in the P105 + EAN binary system is shown in Figure 4. The sharp Bragg peaks are indicative of crystallinity, and they become weaker at high hkl indexes due to long-range disorder. We indicate (with the dotted arrows) the position of the reflections that are expected for the Pm3n space group. The cubic cell lattice parameter obtained from the 1/d_hkl_ versus (h^2^ + k^2^ + l^2^)^1/2^ plot (Figure 3) is 252.7 Å. By using equations reported in ref. [64], we estimated the micelle association number (i.e., the average number of block copolymers in a micelle) to be 65, and an interfacial area of 188 Å^2^, under the assumption that all PPO (and only PPO) formed the core of the micelles. The Pm3n space group has been observed in the systems of surfactants and lipids [65,66,67]. It has also been observed in some PEO-PPO-PEO block copolymer systems: Pluronic P104 + water [68,69], Pluronic P105 + formamide [59], and Pluronic P105 + water + ethanol [64].

### 2.3. Characteristic Scale Lengths of Lyotropic Liquid Crystalline Structures

**Figure 5:**
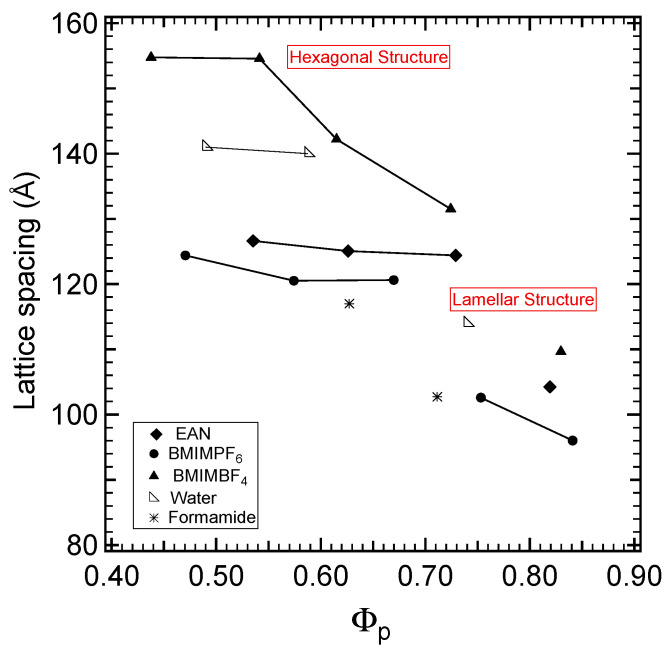
Lattice spacing values of hexagonal and lamellar phases formed in solvent + P105 (EO_37_PO_58_EO_37_) binary systems. The chart is plotted with respect to the volume fraction of the block copolymer in the ionic and molecular solvent systems.

The lattice parameters for the samples in all lyotropic liquid crystalline regions are plotted versus the volume fraction of the block copolymer in Figure 5. For comparison purposes, corresponding data from the P105 (EO_37_PO_58_EO_37_) + water and P105 (EO_37_PO_58_EO_37_) + formamide binary systems are included in Figure 5. The values of the characteristic scale lengths for the hexagonal and lamellar regions of the binary systems comprising Pluronic P105 (EO_37_PO_58_EO_37_) and ionic liquids at various compositions are presented in Table 1.

Figure 5 shows that the lattice spacing for all the binary systems decreases at increasing block copolymer concentrations. The Pluronic P105 self-assembled structures have the highest lattice spacing in ΒΜΙΜΒF_4_, followed by those in EAN and ΒΜΙΜPF_6_. Higher lattice spacing values in the hexagonal mesophase in the binary P105 + BMIMBF_4_ system suggest a smaller number of adjacent cylinders of a greater thickness. The lattice spacing values for the hexagonal and lamellar structures in the different ionic liquids reflect different solvent selectivity. A highly selective solvent results in the formation of microstructures of greater domain spacing. The interfacial area between the block domains decreases so as to reduce the solvophobic interactions between the solvent and the solvophobic blocks. The increased selectivity of a solvent results in microstructures with lower interfacial curvature. The lattice spacing trends suggest that BMIMBF_4_ displays the highest solvent selectivity, followed by EAN and BMIMPF_6_.

**Figure 6:**
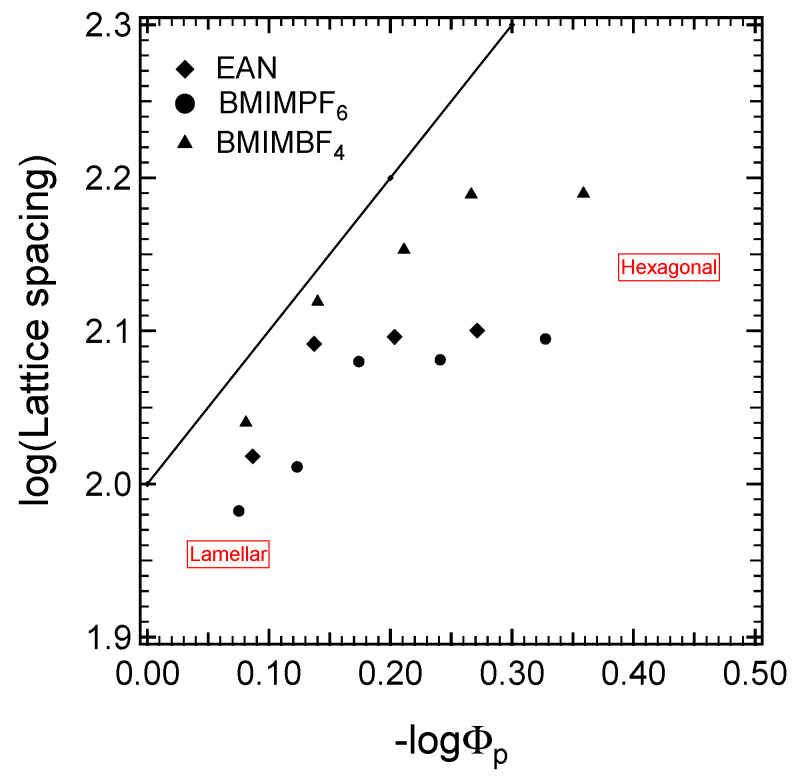
Lattice parameter, d, plotted versus the copolymer volume fraction, 1/Φ_p_. The solid line represents the expected dependence for ideal swelling (d ~Φ_p_^−1^).

The highest selectivity for BMIMBF_4_ can also be inferred by the deviation from the ideal (one-dimensional) swelling behavior demonstrated in Figure 6, where the ideal swelling is represented by the solid line. “Ideal swelling” is when the ratio of the lattice parameter to the amphiphile volume fraction remains constant in the swelling process, and only the interdomain spacing is affected by the addition of solvent. In other words, the solvent does not penetrate into the amphiphile. The exponent of the power law describing the dependence of the lattice parameter on the block copolymer volume fraction is exponent 1 for L_α_ and 0.5 for H_1_ [70]. Such “ideal” swelling behavior has been reported for low-molecular-weight surfactants [71]. Figure 6 shows that the behavior of the PEO-PPO-PEO block copolymer in binary systems with iLs is far from ideal swelling. Both interdomain and intradomain spacing vary during swelling, which implies strong interactions between the copolymer blocks and iLs. The power law exponent expresses the strength of block segregation. The higher the selectivity of the solvent (i.e., the more it swells the PEO domains and the more the solvophobic PPO domains remain unchanged during the dilution of the block copolymer), the closer it is to ideal swelling. The data points for BMIMBF_4_ follow the ideal swelling (higher power law exponent) closer, indicating the higher selectivity of this solvent for PEO compared to the other two ionic liquids studied here.

**Figure 7:**
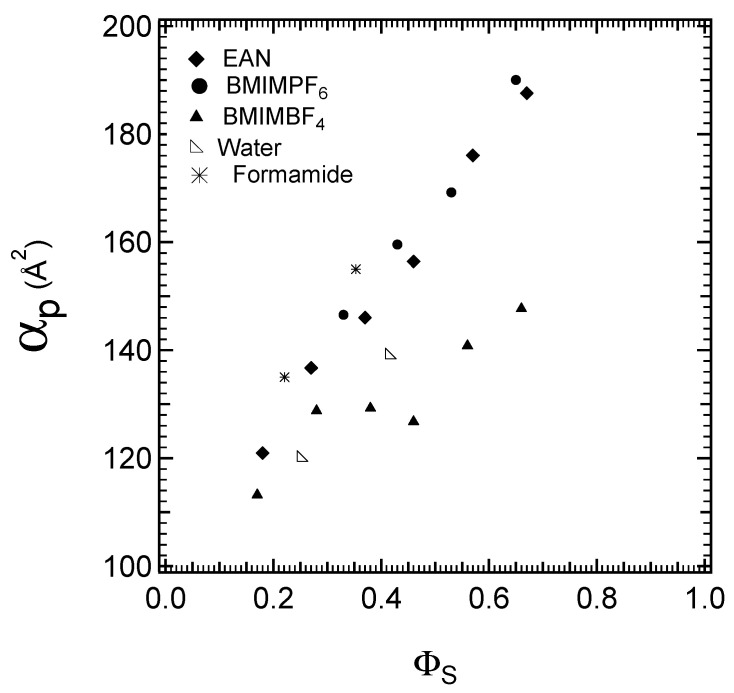
Interfacial areas for the cubic, hexagonal, and lamellar structures observed in binary Pluronic + solvent systems, plotted with respect to the volume fraction of the solvent.

The interfacial areas per PEO block for the samples in all LLC regions, when plotted with respect to the volume fraction of the solvent, are shown in Figure 7; this further demonstrates that the most selective ionic solvent, BMIMBF_4_, has the lowest interfacial area values, indicating strong solvophobic interactions with PPO blocks. In addition, the interfacial area values increase with increasing solvent content due to the swelling of the PEO block as well as the different strengths of the interactions with each of the blocks. Changes in the interfacial area may be captured by a power law α_p_~Φ_p_^(−a)^, where a high exponent value indicates decreased solvent selectivity. In the systems we studied, the highest values of the interfacial area are observed in the EAN and BMIMPF_6_ systems.

**Figure 8:**
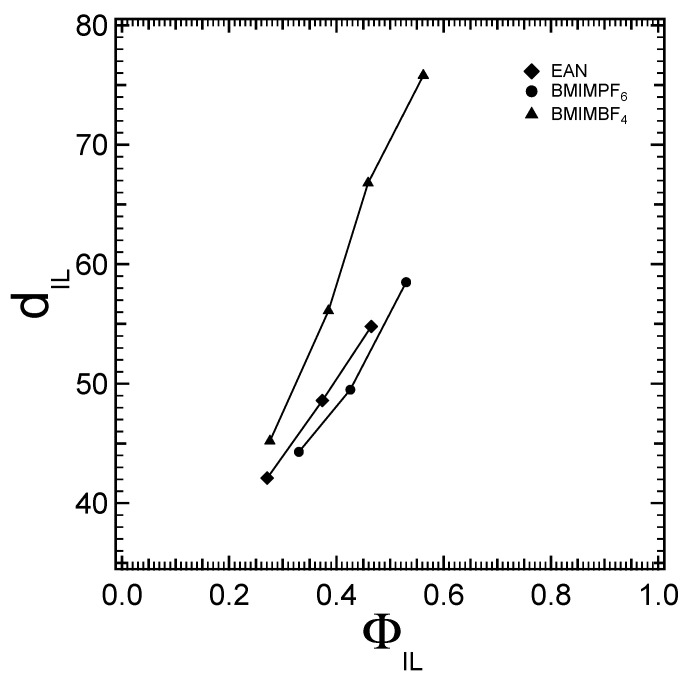
Distance between the cylindrical micelles (thickness of the ionic liquid layer) of the hexagonal structure, plotted with respect to the ionic liquid volume fraction.

In addition to the lattice parameter and interfacial area trends discussed above, the distance between the block copolymer cylinders with respect to the ionic liquid content is illustrated in Figure 8. The system with BMIMBF_4_ exhibits the thickest layer in comparison to the other two ionic liquids, reaffirming the strongest solvophobic interactions with PPO. The longest distance between the exterior of the PPO cylinders observed in the case of BMIMBF_4_ indicates that BMIMBF_4_ is the most selective solvent, and the thickness of the PEO swollen region is higher because of the higher partition of the ionic solvent. Meanwhile, this hydrophobic/solvophobic (PPO) moiety displays the weakest solvophobic interactions with BMIMPF_6_, as illustrated in Figure 8. The thickness of the BMIMPF_6_ layer is the smallest one.

### 2.4. Molecular Interactions Underlying Ionic Liquid Solvent Selectivity

The addition of a selective solvent in a block copolymer changes the interfacial area due to the swelling of the solvated block. Specifically, the interfacial area changes due to interactions between the monomers of the different blocks being replaced by interactions of the selective solvent with each or only one of the blocks [70]. The PEO-PPO interactions in the systems studied here are replaced by PEO-IL and PPO-IL interactions. The ionic liquids considered here are selective solvents for the PEO block and partition to the PEO-rich polar domain of the microstructures. Ionic liquid-PEO interactions in each of the studied systems are illustrated below.

The chemical structure of EAN suggests that solvation is promoted via hydrogen bonding. The hydrogen bonding between the ammonium cations and the nitrate anions leads to the formation of a three-dimensional hydrogen-bonded network similar to the one of water [72]. EAN is a good solvent for PEO [73]. Hence, the PEO moiety of the Pluronic block copolymer is solvated by EAN, promoting its segregation from the PPO block [74]. The ethylammonium cation (CH_3_CH_2_NH_3_^+^), in particular, interacts via H-bonding with the oxygen atom of the PEO group [75]. PPO is reported to be soluble in EAN up to 1 wt.%, with a cloud point of around 34 °C [76]. However, EAN is a less effective solvent for PEO compared to water, as indicated by a more contracted conformation of PEO in EAN (81 Å radius of gyration for 38 kDa PEO) compared to that in water (96 Å) [76].

Both aprotic ionic liquids examined here have an imidazolium ring in the cation. The different anions of the aprotic ionic liquids affect their solvent strength by interacting selectively with certain parts of the amphiphiles. In aprotic ionic liquids, apart from long-range Coulombic interactions, the hydrogen bonding between the anion and the cation is crucial for solvation. During solvation, the H atoms of the imidazolium ring form hydrogen bonds with the solute, which competes with anions [77]. The hydrogen bond acidity (α) of BMIMPF_6_ is higher than BMIMBF_4_, and the hydrogen bond basicity (β) of ΒMIMPF_6_ is lower than BMIMBF_4_. Therefore, the hydrogen bond donor ability of BMIMPF_6_ is expected to be higher [78]. In addition, on the basis of the vibrational spectroscopy results, the cation–anion interaction is stronger in BMIMBF_4_ than in BMIMPF_6_ [79]. Hence, ΒΜΙΜPF_6_ has weaker hydrogen bonds between the ring and the anion that can be disrupted more easily. It is also possible that hydrogen bond interactions may occur between the anion and the PEO groups of the block copolymer. Additionally, it has been shown that the anion, its type, and its size affect electrostatic interactions and, eventually, the segregation strength and the self-assembled morphologies [80].

The solubility of PEO homopolymer has been established in [BMIM]^+^-based ionic liquids [81,82]. Interactions between the oxygen atom of the PEO chains and the H atoms of the imidazolium cations [83] are preferred, despite the fact that hydrogen bonding also forms between the EO hydroxyl group and the fluorine of [PF_6_]^−^ [84]. The experimental results have shown that the imidazolium ring may act as a hydrogen bond donor, whereas the terminal hydroxyl groups and the ethoxy groups of PEO act as hydrogen bond acceptors. In parallel, the [PF_6_]^−^ anion may act as an acceptor, and the terminal hydroxyl groups of PEO may act as donors [85]. The FTIR spectra of the binary systems of PEO-PPO block copolymers and BMIMPF_6_ verified the hydrogen bonding activities with the PEO block [63]. More specifically, Costa et al. [81] studied the molecular interactions between BMIMPF_6_ and PEO by means of molecular dynamics simulations. The study indicated that the ionic structure of BMIMPF_6_ is disrupted during the solvation of PEO; the oxygen atoms on the PEO chains interact with the imidazolium cation, interrupting the cation–anion interactions. Triolo et al. [82] studied BMIMBF_4_-PEO systems employing SANS and concluded that ionic liquid acts as a good solvent and PEO organized itself in random coils.

### 2.5. Comparison of Phase Behavior and Structure in Water or Formamide

It is of interest to compare ionic liquid solvents to molecular solvents in terms of their impact on self-assembly. Overall, the structural polymorphism exhibited by Pluronic P105 in ionic liquids is similar to that reported for the same copolymer in water [4] and in the polar organic solvent formamide [59]. The segregation of the PEO-PPO blocks that is amplified in water due to hydrophobic phenomena [37] is extended in ionic solvents and, hence, is attributed to the solvophobic interactions between PPO and the solvent, with hydrogen bonding remaining the central issue. In all systems, the interfacial areas increase with increasing the solvent content (from L_α_ to H_1_).

The polarity of EAN and the strength of hydrophobic interactions are comparable to what has been reported for water [45]. However, there are indications that the solvophobic interactions in EAN are weaker than in water. In other words, the solvophobic groups display a higher affinity for EAN [45,86]. This is consistent with our observation that the Pluronic P105 LLCs formed in EAN have lower lattice spacing values (Figure 5) and higher interfacial area values (Figure 7) than those in water. The micellar solution phase of Pluronic also suggests the difference in solvophobic interactions: the CMC (critical micellization concentration [36]) values are two orders of magnitude larger in EAN than those in water, and the solvophobicity of PPO for Pluronic F127 (EO_37_PO_58_EO_37_) and Pluronic P123 ((EO_20_PO_70_EO_20_) is lower in EAN than that in water [87].

Aprotic BMIMBF_4_ is the most PEO-selective ionic liquid solvent out of all those investigated in the present study, and it has similar selectivity with water. Conversely, formamide has supported structures with lattice spacing and interfacial values comparable to the ones formed in BMIMPF_6_. In formamide and in BMIMPF_6_, the polymers are less swollen than in water.

Interestingly, the addition of ionic liquids impacts the self-assembly behavior of Pluronic block copolymers in an aqueous solution, as was established in our previous work [22,23,74] and the work of others. Using molecular dynamics (MDs) simulations for Pluronic F68 (EO_103_PO_39_EO_103_) and Pluronic L35 (EO_18_PO_29_EO_18_) aqueous solutions, Pe’rez-Sa’nchez et al. found that the addition of choline-based ILs affected Pluronic micelle formation in a different manner based on different anionic structures [88]. Choline hexanoate ([Ch][Hex]), with a longer alkyl chain of anions compared to choline chloride ([Ch]Cl), was found to promote Pluronic micellization. In the F68 system, [Ch]Cl was arranged around the small aggregates formed (mostly unimers). In the L35 system, [Ch]Cl was located outside of the micelle core, with some around the PEO groups in the micelle corona. On the contrary, with the addition of [Ch][Hex], [Hex]^−^ was arranged well inside the micelle core, whereas [Ch]^+^ was found in the micelle corona in both Pluronic systems. Furthermore, in the [Ch][Hex] mixtures, it was observed that PEO segments twisted towards the micelle core, promoting micelle dehydration and leading the system to a phase separation [88].

### 2.6. Comparison with Other Block Copolymer + Ionic Liquid Binary Systems

Lyotropic phase behavior similar to that reported here has been observed for the low PEO content block copolymer Pluronic P123 (EO_20_PO_70_EO_20_) in EAN [75,89]. A reverse bicontinuous cubic phase (V_2_) has been identified in the P123 + EAN binary system within a narrow concentration range (92–94 EAN wt.%). This phase is not present in water, nor was it identified in our Pluronic P105 systems. The SAXS structural analysis suggested that the lattice spacing values are smaller than those in water [89]. Our observations on the relative ionic solvent selectivity for P105 are in accordance with that reported for P123 + IL. Similar to our findings, the lattice spacing values reported for the anisotropic microstructures are higher for Pluronic P123 in EAN than in BMIMPF_6_ [89,90]. In addition, for Pluronic P123, the values of the characteristic scale lengths in water are higher than the ones in ionic solvents. For the micellar cubic phase, the crystallographic space group Fm3n has been assigned to Pluronic P123 systems, whereas the Pm3n group is suggested to be more appropriate for Pluronic P105 systems. Pluronic P123 is totally immiscible with EOAN (ethanolammonium nitrate) and DEOAF (diethanolammonium formate), which are PILSs with hydroxyls on the alkyl chains and have negligible structural order compared to EAN. These solvents are less polar than EAN and water; hence, they do not favor the solvation of hydrophilic EO groups [91]. The temperature dependence of the phase behavior is found to be modest [75].

A rich variety of block copolymer lyotropic liquid crystals is observed in APIL media. The microphase segregation of high PEO content Pluronic F127 in BMIMPF_6_ is found by using SAXS and polarized optical microscopy [63]. Pluronic P123 forms the same sequence of ordered mesophases in BMIMPF_6_ as that in water and EAN (other than the V_2_ in EAN) while displaying smaller lattice spacing values and negligible temperature dependence [90]. In addition, the formed isotropic micellar phase extends to a larger concentration range than the respective one in water [84]. The ionic liquid n-butyl-n-methylpyrrolidinium bis(trifluoromethanesulfonyl)imide (Pyr_14_TFSI) was found to be a poor solvent for Pluronic F127, with the PPO block being immiscible and the PEO blocks not being well miscible. At high Pluronic concentrations, crystallization was observed for both Pyr_14_TFSI and PEO, forming lamellar and hexagonal structures. It was also observed that the addition of Li ions promotes the solubilization of the PEO chains in the IL, changing from a hexagonal to a lamellar and further to a micellar phase [92,93].

Polybutadiene-PEO diblock copolymer LLCs are reported to form in BMIMPF_6_ and in EMITFSI (1-ethyl-3-methylimidazolium-bis (trifluoromethyl-sulfonyl)imide), with BMIMPF_6_ being the more selective solvent. The structural assignment and phase transitions were verified by means of SAXS and cryo-TEM. The structural scale lengths are smaller than the ones in water [93]. Poly (styrene-block-2-vinylpyridine) (S2VP) copolymers form lyotropic and thermotropic mesophases in imidazolium TFSI ionic liquid, which is selective for the P2VP block [94,95]. Partially sulfonated poly (styrenesulfonate-b-methylbutylene) (S_n_MB_m_) copolymers with different molecular weights and sulfonation levels (SLs) have been investigated in mixtures with imidazolium-based ionic liquids. Lamellar, hexagonal, and spherical structures are obtained. Their curvatures (morphology) depend upon the SLs of the S_n_MB_m_ block copolymers as well as the amount of the incorporated ionic liquid [96].

## 3. Materials and Methods

**Materials.** The Poly(ethylene oxide)-poly (propylene oxide)-Poly(ethylene oxide) (PEO-PPO-PEO) block copolymer Pluronic P105 (EO_37_PO_58_EO_37_) was obtained from BASF Corp. and was used as received. According to the supplier, Pluronic P105 has an average molecular weight of 6500 and 50% PEO content. “Pluronic” refers to products by BASF. Poloxamer is the generic name for PEO-PPO block copolymers.

**Figure 9:**
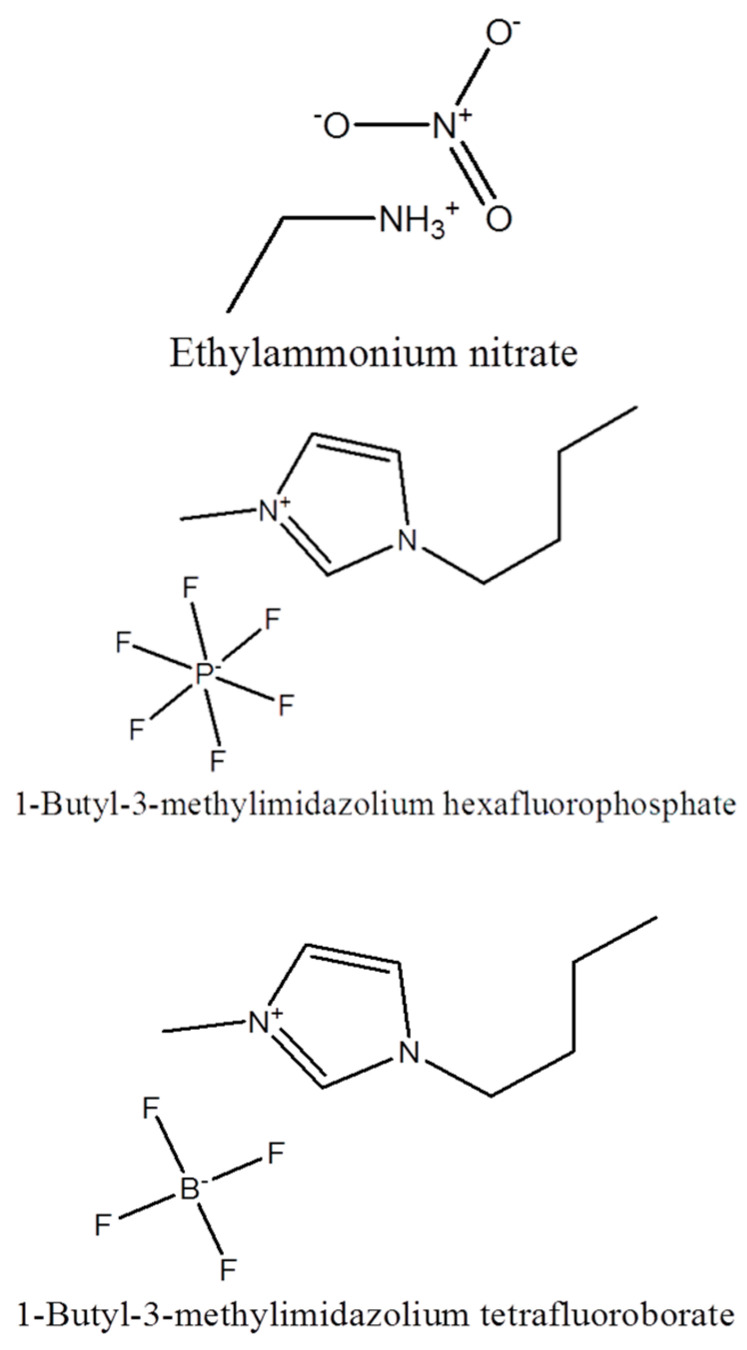
Chemical structures of ethylammonium nitrate (EAN), 1-butyl-3-methylimidazolium hexafluorophosphate (BMIMPF_6_), and 1-butyl-3-methylimidazolium tetrafluoroborate (BMIMBF_4_).

Ethylammonium nitrate (EAN) (CH_3_CH_2_NH_3_^+^NO_3_^−^) was purchased from IoLiTec Ionic Liquids Technologies GmbH (Denzlingen, Germany). The imidazolium-based ILs 1-butyl-3-methylimidazolium hexafluorophosphate (BMIMPF_6_) and 1-butyl-3-methylimidazolium tetrafluoroborate (BMIMBF_4_) were purchased from Sigma Aldrich. The chemical structures of the ILs studied here are shown in Figure 9.

**Sample Preparation.** The ionic liquids were stored in a desiccator to avoid exposure to atmospheric humidity. The samples were prepared individually by weighing the appropriate amount of polymer and the respective amount of each ionic liquid in glass tubes, which were flame-sealed immediately. Subsequently, they were centrifuged repeatedly in both directions over the course of several days in order to facilitate mixing and establish homogeneity [37]. Thereafter, the samples were kept at T = 25 °C and were inspected for phase separation. The single-phase samples are optically clear and completely homogeneous. The phase-separated samples can either be completely opaque or display some distinct heterogeneity indicative of phase separation. The single-phase homogeneous samples were examined by means of polarized light for their optical anisotropy. The micellar solutions and micellar cubic or bicontinuous cubic lyotropic liquid crystalline samples were expected to be isotropic, hence, non-birefringent, whereas the hexagonal and lamellar lyotropic liquid crystals exhibit birefringence/optical anisotropy [37]. All samples characterized herein were transparent, an indication of their homogeneity at the sub-micrometer scale. All samples examined were equilibrated for at least one month before the tests, which is sufficient time for equilibrium.

**Small Angle X-ray Scattering (SAXS).** SAXS experiments were performed at T = 25 °C using a Nano-STAR instrument (Bruker-AXS, Madison, WI, USA) operating at 40 kV and 35 mA. The sample-to-detector distance was 1015 mm. The X-ray wavelength used was 0.1542 nm (Cu Ka). The angular distribution of the scattered electrons was recorded in a two-dimensional detector. The scattering intensity was derived after averaging the intensity of all points in the 2D detector space for a scattering vector value, *q*, defined as

(1) $$q=\frac{4\pi }{\lambda }\sin\left(\frac{\theta }{2}\right)$$

where *θ* is the angle between the incident beam and the scattered radiation.

The structural analysis of the SAXS data was performed by following the procedures discussed by Alexandridis et al. [37]. The relative position of the Bragg peaks is initially assessed by examining the scattering patterns. Further assessment was performed using the Igor Multipeak fitting program (Wavemetrics, Inc., Lake Oswego, OR, USA).

**Microstructure Characterization**. The characteristic scale lengths of the LLCs were obtained from the SAXS diffraction patterns. In the lamellar structure, the lattice spacing is the repeated distance, d, between the planar micelles. Therefore, the lamellar periodicity is given by the following relationship:

(2) $$d=\frac{2\pi }{q^{*}}$$

q* is the position of the first and most intense peak in the SAXS diffraction patterns.

For the hexagonal structure, the lattice parameter is defined as the distance between the planes of the centers of two adjacent rows of cylinders and is defined as

(3) $$a=\frac{4\pi }{q^{*}\sqrt{3}}$$

We define the interfacial area per PEO block as the area that a PEO block of a PEO-PPO-PEO block copolymer occupies at the interface between more polar and less polar domains. The interfacial area for the hexagonal and lamellar structures, assuming that the less polar domains consist of only PPO, are given by the following equations, respectively:

(4) $$a_{p}=\frac{\nu_{p}}{d\cdot \Phi_{p}}$$

(5) ap=νpa·Φp·2π3·f12

*f* stands for the solvophobic (PPO) volume fraction of the specific composition sample of each binary system. For the hexagonal structure, the less polar (hydrophobic part) of the microdomains has a cylinder radius given by the following expression:

(6) R=a·32π·f12

The thickness of the ionic liquid-rich layer is defined as

(7) $$d_{IL}=a-2R$$

For the lamellar structures, the thickness, *δ*, of the less polar (solvophobic) lamellar is given by

(8) δ=fd

The values obtained for the characteristic scale lengths defined above are presented in Table 1.

## 4. Conclusions

The goal of this study was to establish the phase behavior and ordered (lyotropic liquid crystalline) structures in amphiphilic block copolymer + ionic liquid binary systems and probe the interactions between amphiphiles and solvents that underlie self-assembly.

A rich structural polymorphism was observed via the SAXS analysis. The balanced (50% PEO and 50% PPO) block copolymer Pluronic P105 forms positive (“oil” in “water”) curvature LLCs in the ionic liquids EAN, ΒΜΙΜΒF_4_, and BMIMPF_6_ that are selective solvents for PEO. The micellar solution, micellar cubic, hexagonal, bicontinuous cubic, and lamellar LLC phases were observed in all IL solvents considered here, with variations to the compositions of the phase boundaries. For example, the various phases all start to form at higher Pluronic concentrations in EAN than in the imidazolium ILs. When comparing BMIMBF_4_ and BMIMPF_6_, the hexagonal phase has a larger range in BMIMBF_4_ than in BMIMPF_6_.

The behavior of Pluronic P105 in binary systems with ILs is far from ideal swelling, indicating that ILs interact with both PEO and PPO blocks. In the studied ionic liquids, the structures formed by Pluronic P105 have the highest lattice spacing in ΒΜΙΜΒF_4_, followed by those in EAN and ΒΜΙΜPF_6_. The lattice spacing trends suggest that water has a similar selectivity for BMIMBF_4_ for P105, followed by EAN and BMIMPF_6_, which are similar to formamide. It is suggested that the ionic liquid anions determine the solvophilic and solvophobic interactions of the ionic solvent with the block copolymer blocks, eventually resulting in diverse solvent selectivity.

The stability region of the micellar solution phase in ionic liquid solvents is shifted to higher polymer concentrations compared to that in water and formamide. The Pm3n space group, as well as a bicontinuous cubic LLC phase, were identified in all ionic solvents and formamide but were not present in highly selective water. The effective curvature of the microstructures in ionic solvents is higher than in water.

For the first time, this work compares the structural polymorphism and characteristic scale lengths of PEO-PPO-PEO block copolymers in protic ionic liquid, aprotic ionic liquid, and molecular solvents. Such knowledge contributes to the understanding of block copolymer self-assembly in selective solvents. Furthermore, this work provides guidance in the design of ionic liquid-containing complex fluids and soft materials that can be applied in various fields, such as polymer electrolytes [35,53], nanomaterials synthesis [12,97], and formulations for delivery of actives [14,56].

## Figures and Tables

**Table 1:** Characteristic structural scale lengths across the hexagonal and lamellar regions in the P105 (EO_37_PO_58_EO_37_) + EAN, BMIMPF_6_, or BMIMBF_4_ binary systems.

**P105 wt.%**	**EAN wt.%**	**Φ_P105_**	**Φ_EAN_**	**f(PPO)**	**d (Å)**	**α (Å)**	**α_p_(Å^2^)**	**R**	**d_IL_**
0.500	0.500	0.535	0.465	0.289	109.1	125.9	156.4	35.6	54.8
0.592	0.408	0.626	0.374	0.338	108.0	124.8	146.0	38.1	48.6
0.700	0.300	0.729	0.271	0.394	106.9	123.5	136.7	40.7	42.1
0.797	0.203	0.819	0.181	0.442	104.0		120.9	46.0	
**P105 wt.%**	**BMIMPF_6_ wt.%**	**Φ_P105_**	**Φ_BMIMPF6_**	**f(PPO)**	**d (Å)**	**α (Å)**	**α_p_(Å^2^)**	**R**	**d_IL_**
0.403	0.597	0.470	0.530	0.254	107.6	124.2	169.2	32.9	58.5
0.506	0.494	0.574	0.426	0.310	103.3	119.2	159.6	34.9	49.5
0.607	0.393	0.670	0.330	0.362	104.1	120.2	146.5	38.0	44.3
0.699	0.301	0.753	0.247	0.407	102.6				
0.801	0.1	0.841	0.159	0.454	96.0				
**P105 wt.%**	**BMIMBF_4_ wt.%**	**Φ_P105_**	**Φ_BMIMBF4_**	**f(PPO)**	**d (Å)**	**α (Å)**	**α_p_(Å^2^)**	**R**	**d_IL_**
0.403	0.597	0.438	0.562	0.236	134.0	154.8	169.2	39.5	75.8
0.506	0.494	0.541	0.459	0.292	133.8	154.6	157.7	43.9	66.8
0.581	0.419	0.615	0.385	0.332	123.2	142.2	159.6	43.0	56.1
0.695	0.305	0.724	0.276	0.391	113.9	131.5	150.6	43.2	45.2
0.809	0.191	0.830	0.170	0.448	109.7		146.5	49.1	

Φ_P105_: volume fraction of P105; Φ_EAN_: volume fraction of EAN; Φ_BMIMPF6_: volume fraction of BMIMPF_6_; Φ_BMIMBF4_: volume fraction of BMIMBF_4_; f (PPO): volume fraction of the less polar (solvophobic) PPO; d (Å): lattice parameter; α (Å): lattice parameter for hexagonal structure; α_p_ (Å^2^): interfacial area per PEO block; R: cylinder radius of PPO-rich domains; d_IL_: thickness of ionic liquid layer.

## Data Availability

Data available upon reasonable request.
